# Putative Panmixia in Restricted Populations of *Trypanosoma cruzi* Isolated from Wild *Triatoma infestans* in Bolivia

**DOI:** 10.1371/journal.pone.0082269

**Published:** 2013-11-29

**Authors:** Christian Barnabe, Rosio Buitrago, Philippe Bremond, Claudia Aliaga, Renata Salas, Pablo Vidaurre, Claudia Herrera, Frédérique Cerqueira, Marie-France Bosseno, Etienne Waleckx, Simone Frédérique Breniere

**Affiliations:** 1 MIVEGEC (Université de Montpellier 1 et 2 - CNRS 5290 - IRD 224), Maladies Infectieuses et Vecteurs: Ecologie, Génétique, Evolution et Contrôle, Institut de recherche pour le développement (IRD), Representation in Bolivia, La Paz, Bolivia; 2 Instituto Nacional de Laboratorios de Salud (INLASA), Department of Entomology, La Paz, Bolivia; 3 Servicio Departamental de Salud (SEDES) of La Paz, La Paz, Bolivia; 4 Department of Tropical Medicine, Tulane University, School of Public Health and Tropical Medicine, New Orleans, Louisiana, United States of America; 5 Plateforme Génomique Environnementale du Labex Centre "Méditerranéen Environnement Biodiversité", Séquençage – Génotypage, Université Montpellier 2, Montpellier, France; Universidade Federal de Minas Gerais, Brazil

## Abstract

*Trypanosoma cruzi*, the causative agent of Chagas disease, is subdivided into six discrete typing units (DTUs; TcI–TcVI) of which TcI is ubiquitous and genetically highly variable. While clonality is the dominant mode of propagation, recombinant events play a significant evolutive role. Recently, foci of wild *Triatoma infestans* have been described in Bolivia, mainly infected by TcI. Hence, for the first time, we evaluated the level of genetic exchange within TcI natural potentially panmictic populations (single DTU, host, area and sampling time).

Seventy-nine TcI stocks from wild *T. infestans*, belonging to six populations were characterized at eight microsatellite loci. For each population, Hardy-Weinberg equilibrium (HWE), linkage disequilibrium (LD), and presence of repeated multilocus genotypes (MLG) were analyzed by using a total of seven statistics, to test the null hypothesis of panmixia (H_0_).

For three populations, none of the seven statistics allowed to rejecting H_0_; for another one the low size did not allow us to conclude, and for the two others the tests have given contradictory results. Interestingly, apparent panmixia was only observed in very restricted areas, and was not observed when grouping populations distant of only two kilometers or more. Nevertheless it is worth stressing that for the statistic tests of "HWE", in order to minimize the type I error (i. e. incorrect rejection of a true H_0_), we used the Bonferroni correction (BC) known to considerably increase the type II error ( i. e. failure to reject a false H_0_). For the other tests (LD and MLG), we did not use BC and the risk of type II error in these cases was acceptable. Thus, these results should be considered as a good indicator of the existence of panmixia in wild environment but this must be confirmed on larger samples to reduce the risk of type II error.

## Introduction

*Trypanosoma cruzi* is the causative agent of Chagas disease, which affects about eight million people in Latin America, of whom 30–40% either suffers or will develop cardiomyopathy, digestive megasyndromes, or both. Moreover, Chagas disease is becoming an emerging health problem in nonendemic areas because of the increasing number of migrants from endemic areas [1]. The *T. cruzi* species exhibits a very high genetic variability similar to that observed within different species of other kinetoplastidae such as *Leishmania* [2]. Consensual taxonomy recognized six discrete typing units (DTUs named TcI–TcVI) [3] and one additional group only found in bats (Tcbat) [4] within *T. cruzi* [5]; TcI is the most genetically diversified and ubiquitous of them, spreading from the United States to Argentina, and present in both sylvatic and domestic biotopes. As a result of the dominant clonal multiplication, identical multilocus genotypes (MLGs) have been sampled over several years and over large geographical distances, leading to considering the species as multiclonal [6]. The long-term clonal evolution is involved in the current important genetic diversity of the species, but more and more “genetic exchange” events are being described. Scarce hybridization events are the source of two hybrid DTUs [7–9], mitochondrial introgression events have been detected [10,11], and different levels of gene recombination have been described [12–14]. In addition, high genome plasticity is also a source of variability. Aneuploidy is suspected [15], occurrence of allele loss is possible during genetic exchanges, the mitochondrial genome is probably more complex than previously described, and maxicircle gene recombination occurs as well as intragenic recombination [14]; heteroplasmy has also been reported [16]. Several of these genetic exchange mechanisms have been triggered in vitro [17] and are still hotly debated in the field. As previously stated [18]: “From an epidemiological and medical point of view, the important parameter to evaluate is the stability of the genetic clones in space and time.” This stability directly depends on the level of genetic exchanges (in the broad sense). Indeed, within a strict clonal framework the clones are stable in space and time, and they convey similar biological characteristics that can be crucial for epidemiological and medical features generation after generation. In contrast, with more or less frequent recombination, such correlations are not necessarily expected, hence the importance of studying genetic exchanges between stocks.

In general terms, to test panmixia, two prerequisites are needed: (i) the use of an appropriate genetic marker not subjected to selection and with a sufficient level of polymorphism and (ii) populations isolated in restricted areas where parasites are assumed to be in sympatry. Our previous work showed that microsatellite markers are relevant for studying the population genetics of *T. cruzi* at the DTU level [19]. Moreover, abundant and accessible foci of wild *Triatoma infestans* vectors mainly infected by TcI have been recently described in Bolivia [20,21]; hence, in the present work it was possible to evaluate the level of genetic exchanges in potentially panmictic *T. cruzi* TcI populations isolated from sylvatic *T. infestans* in Bolivia.

## Materials and Methods

### Parasite stocks and multilocus microsatellite typing (MLMT)

**Figure 1:**
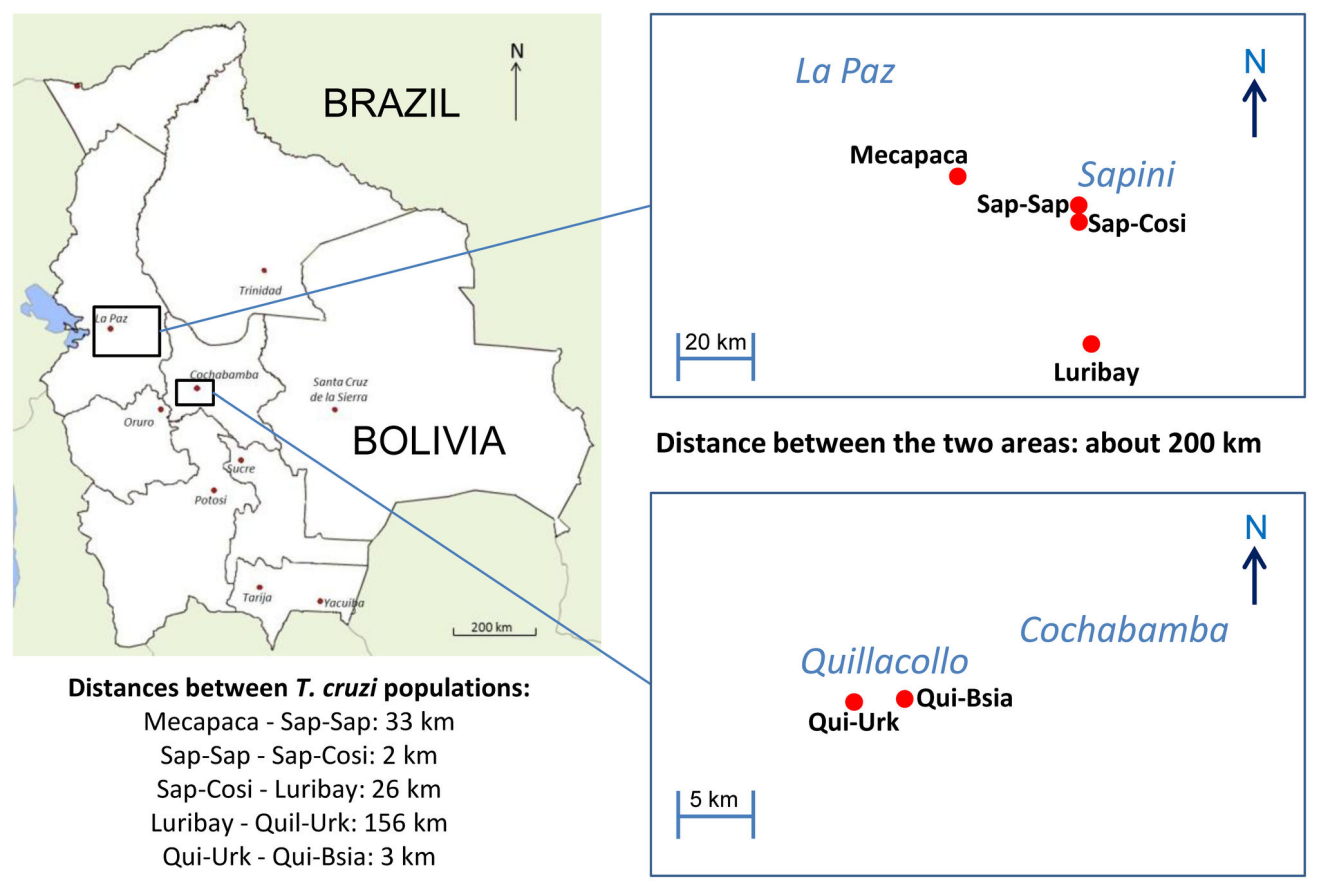
Map of Bolivia: localization of the six populations of *Trypanosoma cruzi* under study isolated from sylvatic *Triatoma infestans* and distances between populations.

Seventy-nine *T. cruzi* stocks, previously assigned to the DTU TcI using the multiplex miniexon PCR method [22] and isolated from six potentially panmictic Bolivian sylvatic *T. infestans* populations (see Figure 1) were compared to 21 TcI sylvatic reference stocks ranging from the United States to South America (see Table 1). These populations were defined in small geographic areas in which we believe that the *T. infestans* vector can move freely (maximum distance between two stocks less than five hundred meters). Four of them are located in La Paz department (namely, Luribay, central sampling point at 17°3'54.90"S / 67°39'53.85"W; Mecapaca, 16°42'45.90"S / 67°59'27.13"W; Sap-Sap, 16°48'47.23"S / 67°42'9.83"W; and Sap-Cosi, 16°49'50.00"S / 67°42'22.20"W), while the other two populations are located in Cochabamba department (namely, Qui-Urk, 17°25'29.00"S / 66°17'45.20"W and Qui-Bsia, 17°25'28.81"S / 66°15'52.75"W). The distances between the populations are given in Figure 1. The stocks directly isolated from wild triatomines, all captured with mice bait Noireau’s traps, were cultured in LIT medium supplemented with 10% fetal calf serum. DNA was extracted with a conventional CTBA 2% method and the solutions diluted to 20 ng/µl before use. Eight previously described microsatellite loci were used, namely MCLE01, SCLE10, SCLE11, MCLF10, A427, MCLG10, C875, and MCLE08 [17,23] using the same PCR conditions [19]. Electrophoreses of fluorescent-labeled PCR products, diluted and denatured in 20 µl of HiDi formamide, were carried out on a ABI3130xL Genetic Analyzer (Applied Biosystems, Carlsbad, CA, USA), with Genescan 500 LIZ as the internal size standard. GeneMapper^®^ software (Applied Biosystems, Carlsbad, CA, USA) was used to characterize the alleles.

**Table 1:** Codes, locations, genotypes at each locus and reference numbers of each multilocus genotype (MLG) of the 79 *Trypanosoma cruzi* TcI stocks isolated from six potentially panmictic populations and of the 21 *T. cruzi* TcI reference strains.

Stock code	Location*	MCLE01		SCLE10		SCLE11		MCLF10		A427		MCLG10		C875		MCLE08		MLG
	LaPaz / Mecapaca / Tun1 / Mecapaca																	
MEC095	id.	128	128	250	254	138	138	188	188	185	185	155	155	191	191	117	117	66
MEC099	id.	128	128	250	254	138	138	188	188	186	186	155	155	183	187	117	117	74
MEC101	id.	128	128	250	254	138	138	188	188	186	186	155	155	183	187	117	119	73
MEC102	id.	128	128	250	254	138	138	184	188	186	186	155	155	183	187	117	119	71
MEC103	id.	128	129	250	254	139	139	184	188	186	186	155	155	191	191	117	117	76
MEC107	id.	128	128	254	254	138	138	184	188	186	186	155	155	191	191	117	119	70
MEC161	id.	128	128	250	254	139	139	188	188	186	186	155	155	187	191	119	119	86
MEC166	id.	128	128	250	254	139	139	188	188	186	186	155	155	187	191	119	119	86
MEC170	id.	128	128	250	254	139	139	188	188	186	186	155	155	183	187	119	119	85
MEC171	id.	127	127	250	250	138	138	184	188	178	184	155	155	183	191	117	119	58
MEC173	id.	128	128	250	250	139	141	184	188	177	184	155	155	183	191	119	119	59
	LaPaz / Luribay / Luribay / Luribay																	
LUR229	id.	128	128	238	250	138	138	184	184	178	186	155	155	189	189	117	117	67
LUR237	id.	128	128	250	250	138	138	184	184	185	185	155	155	189	189	117	117	68
LUR245	id.	120	128	250	254	138	138	184	188	186	186	146	155	189	189	117	117	64
LUR250	id.	128	128	250	254	138	138	184	188	185	185	155	155	189	189	0	0	65
LUR258	id.	128	128	250	250	138	140	184	184	179	186	155	155	189	189	117	117	69
LUR265	id.	128	128	250	250	138	138	184	184	177	187	155	155	187	187	0	0	60
	LaPaz / Murillo / Sapini / Sap-Sap																	
SAP203	id.	129	129	250	254	138	138	184	188	175	178	155	155	187	189	117	119	21
SAP207	id.	128	128	250	254	138	138	188	188	178	178	155	155	187	189	117	119	48
SAP223	id.	120	128	250	254	138	138	184	188	175	178	155	155	187	189	117	119	53
SAP233	id.	129	129	250	254	138	138	184	188	175	178	155	155	189	191	117	119	22
SAP241	id.	117	128	250	254	138	138	184	188	177	177	146	146	187	189	117	119	50
SAP242	id.	129	129	250	254	138	138	184	188	175	178	155	155	187	189	117	119	21
SAP242b	id.	129	129	250	254	138	138	184	188	178	178	146	155	187	189	117	119	24
SAP243	id.	129	129	250	250	138	140	188	188	178	178	155	155	187	189	117	119	35
SAP256	id.	128	128	250	254	138	138	182	186	175	178	146	146	187	189	117	119	51
SAP259	id.	128	128	250	254	139	139	184	188	175	178	146	155	187	189	117	119	52
SAP260	id.	129	129	250	254	138	138	184	188	175	175	146	146	187	189	117	119	26
SAP261	id.	129	129	250	254	138	138	184	188	175	175	146	155	187	189	117	119	25
SAP263	id.	129	129	250	254	138	138	184	188	175	175	155	155	189	191	117	119	23
SAP264	id.	129	129	250	254	138	138	184	184	178	178	155	155	189	189	119	119	19
SAP265	id.	129	129	250	254	138	138	184	188	175	184	155	155	187	187	117	119	30
SAP266	id.	129	129	254	254	138	138	188	188	178	178	155	155	189	189	117	119	17
SAP267	id.	120	128	250	254	138	138	184	188	175	178	146	155	187	189	117	119	54
SAP270	id.	128	128	250	254	138	138	184	188	175	178	155	155	187	189	117	119	55
SAP271	id.	129	129	250	250	138	140	184	188	186	186	146	155	187	189	117	119	39
SAP272	id.	129	129	250	254	138	138	184	188	175	186	146	146	187	189	117	119	27
SAP391	id.	120	128	250	250	138	138	184	184	177	177	155	155	187	187	119	119	62
SAP404	id.	128	128	250	254	138	138	184	188	175	175	155	155	187	189	117	119	56
SAP405	id.	117	128	250	250	138	138	184	184	177	177	155	155	187	187	119	119	63
SAP445	id.	120	128	250	254	138	138	184	184	177	177	155	155	187	187	119	119	61
SAP491	id.	120	128	250	254	138	138	184	188	175	175	155	155	187	189	117	119	57
SAP492	id.	120	128	250	254	138	138	184	188	175	175	155	155	187	189	117	119	57
SAP500	id.	128	128	250	254	138	138	184	188	175	175	155	155	187	189	117	119	56
	LaPaz / Loayza / Cosiraya / Sap-Cosi																	
SAP302	id.	129	129	250	250	137	137	188	188	178	186	155	155	187	187	117	119	37
SAP303	id.	128	128	250	254	137	137	188	188	178	178	155	155	187	187	117	119	49
SAP304	id.	129	129	250	250	138	138	184	188	178	186	155	155	187	189	117	119	34
SAP310	id.	129	129	250	254	138	138	184	188	186	186	146	155	187	187	117	119	28
SAP312	id.	129	129	254	254	138	138	188	188	186	186	146	146	189	189	117	119	18
SAP313	id.	129	129	250	254	138	138	184	188	178	186	155	155	187	187	117	119	31
SAP318	id.	129	129	250	250	138	138	188	188	178	186	146	146	187	187	117	119	46
SAP319	id.	129	129	250	250	138	138	188	188	178	178	146	146	187	187	117	119	45
SAP321	id.	129	129	250	250	138	140	178	188	179	186	146	155	187	187	117	119	40
SAP323	id.	129	129	250	254	138	138	184	188	179	186	155	155	187	187	117	117	32
SAP334	id.	129	129	250	250	138	140	178	178	186	186	155	155	187	187	111	117	41
SAP336	id.	129	129	250	250	138	138	178	178	178	186	146	155	187	187	117	119	43
SAP337	id.	129	129	250	250	138	138	178	178	178	186	146	146	187	187	117	119	44
SAP346	id.	129	129	250	250	138	138	184	188	179	186	155	155	187	187	117	117	33
SAP347	id.	129	129	250	250	138	138	188	188	178	186	155	155	187	189	117	119	36
SAP348	id.	129	129	250	250	137	137	188	188	178	186	146	155	187	189	117	119	38
SAP349	id.	129	129	250	250	138	138	188	188	178	186	146	146	187	187	117	117	47
SAP372	id.	129	129	250	250	138	138	184	184	178	186	146	155	189	189	117	119	20
SAP374	id.	129	129	250	250	138	138	184	188	179	179	146	155	187	187	117	119	42
	Cochabamba / Quillacollo / VillaUrkipiña / Qui-Urk																	
QUI755	id.	129	129	250	254	138	138	188	188	186	186	155	155	187	187	117	119	29
QUI757	id.	129	129	250	254	139	139	188	188	186	186	155	155	187	187	119	119	88
QUI762	id.	120	128	250	254	139	139	188	188	186	186	155	155	187	187	119	119	84
QUI763	id.	129	129	250	254	139	139	188	188	186	186	155	155	187	187	117	119	89
QUI766	id.	129	129	250	254	139	139	188	188	186	186	155	155	187	187	117	119	89
QUI768	id.	129	129	250	254	138	138	188	188	186	186	155	155	187	187	117	119	29
QUI769	id.	120	128	250	250	139	139	188	188	186	186	155	155	187	187	117	119	83
QUI774	id.	129	129	250	254	139	139	188	188	186	186	155	155	187	187	117	119	89
QUI775	id.	129	129	250	254	139	139	188	188	186	186	155	155	187	187	117	119	89
QUI907	id.	129	129	250	254	139	139	188	188	186	186	155	155	187	187	117	119	89
QUI913	id.	129	129	250	254	139	139	188	188	186	186	155	155	187	187	119	119	88
QUI916	id.	129	129	250	254	139	139	188	188	186	186	155	155	187	187	117	117	87
	Cochabamba / Quillacollo / BSIA14T1 / Qui-Bsia																	
QUI026	id.	128	128	250	254	138	138	188	188	186	186	155	155	187	187	117	117	75
QUI027	id.	128	128	250	254	138	138	188	188	186	186	155	155	187	187	117	117	75
QUI053	id.	131	131	250	254	136	136	188	188	186	186	146	155	187	187	117	117	80
QUI054	id.	117	125	250	254	130	140	188	188	186	186	155	155	187	187	117	117	81
	Countries of reference strains																	
361-TA	Colombia	131	133	252	254	135	138	186	186	174	174	157	157	176	182	119	119	7
458	Colombia	131	131	248	251	138	140	186	186	178	178	153	155	172	176	114	119	2
85/818	Bolivia	123	141	251	251	138	138	174	174	177	181	146	155	174	174	119	119	1
93041401P	USA	135	141	255	255	135	135	174	184	188	188	157	157	165	165	114	114	11
93070103P	USA	135	141	255	255	135	135	174	174	188	188	157	157	165	165	114	117	12
A269	Guiana	143	145	251	251	140	140	184	184	179	179	155	157	178	184	114	114	4
Cuicacl1	Brazil	128	128	250	254	139	139	188	188	186	186	146	155	187	187	117	119	82
Cutiacl1	Brazil	129	129	254	254	139	139	178	188	173	186	155	155	165	165	117	117	77
FX18	Colombia	127	131	255	255	136	136	178	178	173	173	159	161	166	185	114	114	5
G-38-1	Brazil	129	129	254	254	138	138	182	182	177	177	153	153	172	172	117	117	15
H10	Mexico	135	137	252	255	135	135	176	176	173	173	157	157	165	165	117	119	9
OPS21cl11	Venezuela	135	135	252	255	135	135	184	186	173	186	157	157	165	165	117	119	10
P209cl93	Bolivia	129	129	238	254	138	138	178	178	186	186	155	155	165	165	117	119	16
PB3cl2	Bolivia	127	155	252	254	138	140	186	188	178	178	155	155	170	170	114	117	3
PERU	Peru	127	127	252	255	128	128	184	186	182	182	149	157	165	165	114	119	8
SABP3	Peru	128	128	250	254	138	138	188	188	186	186	155	155	187	187	119	119	72
Saimiri4A	Venezuela	142	144	251	251	136	136	191	191	178	178	157	157	185	189	111	117	6
SP31	Chile	128	128	254	254	139	139	184	188	173	186	155	155	165	165	119	119	78
T.cruzi#1	Honduras	134	134	252	252	135	135	184	184	0	0	157	157	165	165	119	119	13
V120	Chile	128	128	254	254	139	139	188	188	173	186	155	155	165	169	114	119	79
Z17	Mexico	137	137	255	255	135	135	184	184	175	175	157	157	165	165	119	119	14

^*^ Department / Municipality / Area / Population

### Data analysis

“For a majority of pathogens, including the Trypanosomatidae family, the reproductive strategy was mainly deduced from population genetics analysis” [24]. Here, the analyses were focused on two kinds of events involved in sexual exchanges: allelic segregation and genetic recombination. Allelic segregation was explored through Hardy-Weinberg equilibrium (HWE) or *F*_is_, while genetic recombination was explored through linkage disequilibrium analysis (LD, nonrandom association between genotypes at independent loci) and the presence / absence of repeated multilocus genotypes (MLG). A previous study, based on simulations and aiming to estimate the level of clonal reproduction in diploids [25] advised the simultaneous use of *F*_is_ (mean and variance) and LD estimators.

*F*_is_ is a measure of inbreeding of individuals within a subsample; it also represents the deviation from random union of gametes and varies from −1 (fixed heterozygous) to +1 (fixed homozygous) via *F*_is_ = 0 (Hardy-Weinberg equilibrium). This Wright F-statistic [26] was estimated with Weir and Cockerham’s unbiased estimators [27] called *f*. Negative values of *F*_is_ (excess heterozygosity) can be caused by accumulation of mutations in an ancient clonal lineage, a phenomenon called the Meselson effect [28], and are generally regarded as a mark of clonality as observed in Bdelloid rotifers [29]. Positive values of *F*_is_ correspond to inbreeding within the sample, a particular case being the Wahlund effect, when the sample comes from heterogeneous and structured populations. It is worth noting that if the mean *F*_is_ values are good estimators of HWE, low *F*_is_ values associated with substantial variance of *F*_is_ among loci (with some loci displaying an extreme heterozygote deficit and others an extreme excess) can reveal very low levels of sex (cryptic sex) [30]. All statistical tests were based on randomization: data sets fitting the null hypothesis (H_0_ = panmixia) were generated by randomizing the relevant unit (allele, genotype, etc.). Here, to test HWE within the subsamples, the alleles were permuted among individuals within each subsample and *F*_is_ was used as a HWE estimator, while for testing the overall HWE, alleles were permuted among subsamples and *F*_it_ was used as an estimator. Moreover, since the presence of null alleles artificially increases *F*_is_ estimations, we tested the impact of null alleles on the increased *F*_is_ values.

Linkage disequilibrium (LD) is another measure of deviation from panmixia. Here it was estimated in three different ways: (i) by the classical index I_A_ [31], which has the disadvantage of increasing with the number of loci, so we also used a slightly modified index (ȓ_d_) which is independent of the number of loci [32]; (ii) by the log-likelihood ratio *G*-statistics [33]; the *P*-value of this test is obtained as follows: genotypes at the 2 loci are associated at random a number of times and the statistic is recalculated on the randomized data set; the *P*-value is estimated as the proportion of statistics from randomized data sets that are larger or equal to the observed and (iii) by comparing the observed number of MLGs and the frequency of the most frequent MLG to the expected ones in simulated panmixia. As for *F*_is_, all the LD statistical tests are based on H_0_ = panmixia (i.e., the genotypes at the two loci are associated at random a number of times depending on the sample size and the statistics are recalculated on the randomized data set).

LD and HWE tests are based on multiple comparisons, so the Bonferroni corrections should be applied; this consists in dividing the *p*-value (or α, generally 5%), which is the threshold for rejecting H_0_, by the number of comparisons. For example, testing eight loci within seven different populations leads to 56 comparisons and theoretically α (0.05) would become α′ = α / 56 = 0.00089. Nevertheless, the Bonferroni correction entails a high risk of falsely accepting H_0_ (bias towards Type II error) and therefore masking real deviations from panmixia. Teriokhin et al. [34] suggested that a high test power can be preserved by using the binomial test instead of the Bonferroni correction in order to check whether the proportion of tests found significant at the 5% level was significantly above 0.05: if this is true, the test is significant and H_0_ is rejected, and if it is not true, H_0_ is not rejected ; for example here with 8 loci, to test the genotype association at two loci by using G-statistics there are 26 comparisons and hence 26 values of G-Statistics: if 3 of them are below 0.05 the binomial test (written in R “binom.test (3, 26, p=0.05)”) give a no significant *P*-value of 0.1386, meaning that 3 values under 0.05 out of 26 are not sufficient to reject Ho; in reality we need 4 values below 0.05 out of 26 to reject significantly H_0_ (*P*-value of the binomial test in this case is 0.03874). Because rejecting or accepting the null hypothesis is crucial here, we chose to use and discuss all the *p*-values (with or without the Bonferroni corrections and the *p*-values given by the exact binomial tests).

To test *F*_is_, LD, and MLG, we examined nine subsamples: the six populations under study (Luribay, Mecapaca, Sap-Sap, Sap-Cosi, Qui-Urk, and Qui-Bsia); the subsample “overall Sapini,” which clusters the two populations from Sapini (Sap-Sap + Sap-Cosi); the subsample “overall Quillacollo,” which clusters the two populations from Quillacollo (Qui-Urk + Qui-Bsia); and the “overall” sample including all stocks (*N* = 79). The different indices and *p*-values were associated with their level of significance (NS, not significant; * significant at 5% and ** significant at 1%). As several tests were applied for *F*_is_, LD, and repeated MLG, a decision about accepting or rejecting H_0_ is proposed in each case, namely “reject H_0_” or “not reject H_0_” when all tests are congruent, and “ambiguous” when at least one of the tests gave a discordant result.

To process the data, different programs were used: (i) the HierFstat package [35] in R [36] to compute the 95% confidence intervals of *F*_is_, (ii) the “binom.test” function in R to test the null hypothesis about the probability of success in Bernoulli’s experiments, (iii) MicroChecker v.2.2.3 [37] to test the load of null alleles, (iv) Multilocus v1.3b [32] for I_A_ and ȓ_d_ indices and to test the probabilities of repeated MLG and different MLG, (v) Populations (v.1.2.30^©^ 1999, Olivier Langella, CNRS UPR9034) to build a general clustering analysis between all stocks using the Cavalli-Sforza and Edwards’ chord genetic distances [38], and (vi) Fstat [39] for all other tests.

## Results

### Genetic diversity of the six populations under study

Genetic diversity was explored within the six local wild *T. cruzi* TcI populations (79 stocks) and within the 21 reference strains. Details of the origin and allelic microsatellite composition of each stock studied are listed in Table 1.

Null alleles: Only two stocks from the Luribay population did not amplify at locus MCL08 and one reference stock at locus A427. Analyzing the six potentially panmictic populations with MicroChecker, 43 null alleles were expected at loci presenting high *F*_is_ over 1264 alleles, hence 3.40%, which is already very low. The proportion of observed null alleles in this sample (*n* = 4, hence 0.32%) is lower than expected (exact binomial test, *p* = 4e-14). Thus, the role of null alleles in inflated *F*_is_ may be considered here as negligible.

Overall polymorphism: The main indices of genetic diversity as well as observed and expected heterozygotes and *F*_is_ by locus and by population are listed in Table 2. It is worth noting that, as expected, the subsample of the reference strains (*n* = 21) is by far the most polymorphic. Moreover, 42 alleles out of 82 (51.2% of the total number of alleles) were specific to reference strains (see Table 1). Eighty-nine different multilocus genotypes (MLGs) were observed among the 100 stocks (including references) versus only 68 MLGs among the 79 stocks under study (without references). The most repeated MLG (no. 89, repeated five times) was identified in a single population, Qui-Urk, in the Cochabamba valley (Table 1). The number of alleles per locus ranged from 4 to 18 and from 2 to 8 with and without references, respectively. Similarly, the mean allelic richness by locus systematically decreased when reference strains were removed. For the six local populations, the *F*_is_ values per locus and per population showed high variance, ranging from −1.00 (fixed heterozygosity for locus SCLE10 in Qui-Bsia population) to 1.00 (fixed homozygosity for loci MCLE01 in Sap-Cosi, SCLE11 in Qui-Urk, and C875 in Luribay), while only positive *F*_is_ values were observed for the reference population (ranging from 0.30 to 0.82) as is expected when pooling differentiated reproductive units within a single subpopulation [25]. The mean allelic richness in local populations was weakly variable, ranging from 1.49 (Qui-Urk) to 2.27 (Sap-Sap) and higher within the reference strains (4.49). The clustering analysis (NJ tree not shown) of all the stocks using the Cavalli-Sforza and Edwards distance method showed that six of the reference strains, namely P209cl93, SABP3, Cutiacl1, SP31, V120, and Cuicacl1, were closely related to some of the wild stocks under study, the other reference strains forming a separate group not supported by a significant bootstrap value. The analysis of genetic distances between each of the 21 reference strains and the 79 wild stocks (mean of pairwise distances) showed that the three reference strains closest to the Bolivian wild stocks were SABP3 from Peru, Cuicacl1 from Brazil, and P209cl93 from Bolivia, with genetic distances of 0.36, 0.41, and 0.50, respectively; the three reference strains farthest from the wild stocks were FX18 from Colombia and 93041401P and 93070103P from the US, with mean genetic distances of 0.89, 0.88, and 0.87, respectively.

**Table 2:** Main indices of genetic diversity and Hardy-Weinberg equilibrium by locus (vertical) and by population (horizontal) of the 79 *Trypanosoma cruzi* TcI stocks isolated from six potentially panmictic populations and of the 21 *T. cruzi* TcI reference strains.

Population		MCLE01	SCLE10	SCLE11	MCLF10	A427	MCLG10	C875	MCLE08	Overall
Luribay	N/no. all/all. rich.	6/2/1.67	6/3/2.58	6/2/1.67	6/2/1.91	6/6/4.66	6/2/1.67	6/2/1.91	4/1/1.00	2.13*
	H_o_/H_e_/*F*_is_	0.17/0.17/0.00	0.50/0.44/-0.15	0.17/0.17/0.00	0.33/0.30/-0.11	0.50/0.82/0.41	0.17/0.17/0.00	0.00/0.30/1.00	NA	0.23/0.30/0.24
	MLG	-	-	-	-	-	-	-	-	6
Mecapaca	N/no. all/all. rich.	11/3/1.97	11/2/2.00	11/3/2.36	11/2/1.92	11/5/2.94	11/1/1.00	11/3/2.92	11/2/2.00	2.14*
	H_o_/H_e_/*F*_is_	0.09/0.25/0.65	0.73/0.52/-0.43	0.09/0.56/0.84	0.45/0.37/-0.25	0.18/0.47/0.63	NA	0.73/0.67/-0.08	0.36/0.52/0.31	0.33/0.42/0.23
	MLG	-	-	-	-	-	-	-	-	10
Qui-Urk	N/no. all/all. rich.	12/3/2.13	12/2/2.00	12/2/1.83	12/1/1.00	12/1/1.00	12/1/1.00	12/1/1.00	12/2/1.97	1.49*
	H_o_/H_e_/*F*_is_	0.17/0.30/0.46	0.92/0.52/-0.83	0.00/0.29/1.00	NA	NA	NA	NA	0.67/0.50/-0.33	0.22/0.20/-0.08
	MLG	-	-	-	-	-	-	-	-	6
Qui-BSIA	N/no. all/all. rich.	4/4/4.00	4/2/2.00	4/4/4.00	4/1/1.00	4/1/1.00	4/2/2.00	4/1/1.00	4/1/1.00	2.00*
	H_o_/H_e_/*F*_is_	0.25/0.75/0.70	1.00/0.57/-1.00	0.25/0.75/0.70	NA	NA	0.25/0.25/0.00	NA	NA	0.22/0.29/0.28
	MLG	-	-	-	-	-	-	-	-	3
Sap-Sap	N/no. all/all. rich.	27/4/2.89	27/2/1.99	27/3/1.55	27/4/2.29	27/5/3.25	27/2/1.91	27/3/2.27	27/2/1.99	2.27*
	H_o_/H_e_/*F*_is_	0.30/0.63/0.53	0.81/0.50/-0.64	0.07/0.14/0.48	0.74/0.54/-0.37	0.37/0.68/0.46	0.18/0.37/0.51	0.78/0.54/-0.44	0.85/0.50/-0.73	0.51/0.49/-0.05
	MLG	-	-	-	-	-	-	-	-	24
Sap-Cosi	N/no. all/all. rich.	19/2/1.38	19/2/1.78	19/3/2.17	19/3/2.72	19/3/2.70	19/2/1.99	19/2/1.84	19/3/2.20	2.10*
	H_o_/H_e_/*F*_is_	0.00/0.10/1.00	0.21/0.27/0.23	0.10/0.36/0.71	0.37/0.57/0.36	0.68/0.61/-0.12	0.31/0.50/0.38	0.16/0.31/0.49	0.84/0.52/-0.64	0.33/0.41/0.18
	MLG	-	-	-	-	-	-	-	-	19
REF	N/no. all/all. rich.	21/15/6.07	21/7/4.23	21/6/4.26	21/8/4.98	20/10/5.31	21/7/3.38	21/13/4.60	21/4/3.05	4.49*
	H_o_/H_e_/*F*_is_	0.43/0.91/0.54	0.43/0.80/0.47	0.14/0.80/0.82	0.29/0.85/0.67	0.25/0.87/0.72	0.29/0.68/0.58	0.29/0.74/0.62	0.48/0.67/0.30	0.32/0.79/0.60
	MLG	-	-	-	-	-	-	-	-	21
Overall with reference strains (*N* = 100) / without reference strains (*N* = 79)										
	no. all	18/7	7/3	9/7	8/5	13/8	7/2	15/4	4/3	-
	Size range**	117-155	238-255	128-141	174-191	173-188	146-161	165-191	111-119	-
	mean all. rich.	3.64/2.75	2.96/2.02	3.19/2.58	3.08/2.37	4.24/3.75	2.54/1.83	3.70/2.67	2.44/2.04	-
	*F*_is_	0.56/0.57	-0.17/-0.49	0.76/0.72	0.20/-0.07	0.44/0.31	0.48/0.41	0.17/-0.07	-0.24/-0.46	-
	H_s_	0.46/0.38	0.51/0.45	0.45/0.39	0.39/0.30	0.51/0.44	0.29/0.22	0.38/0.31	0.39/0.34	-

N, size of the sample; no. all, number of alleles; all. rich., allelic richness; H_o_, observed heterozygotes; H_e_, expected heterozygotes; MLG, number of different multilocus genotypes in the subsample; REF, reference stocks; H_s_, Nei's gene diversity; *, mean allelic richness; **Size range of alleles calculated with all stocks (including references); NA, not available because the locus is monomorphic.

### Panmixia tests within the six populations under study

**Figure 2:**
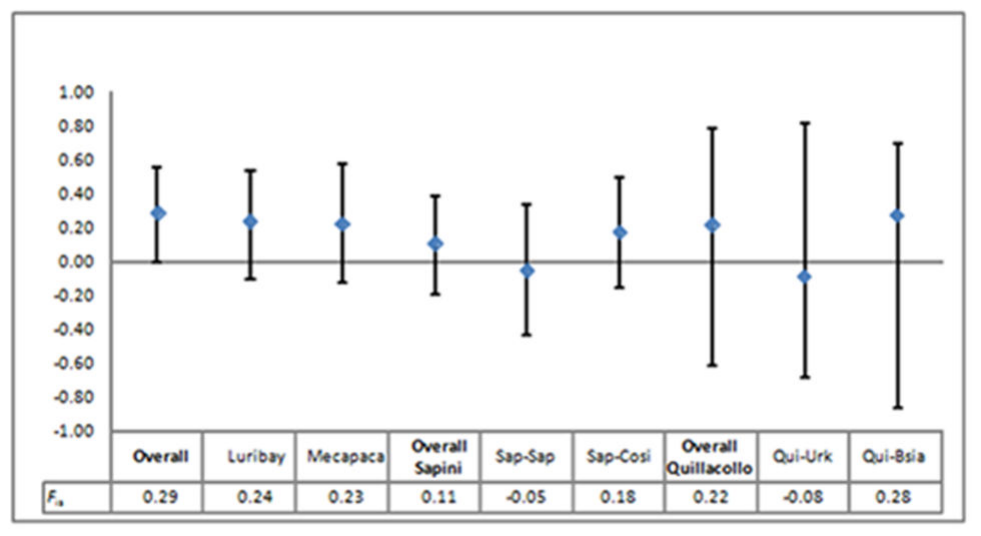
Observed *F*_is_ of the six *Trypanosoma cruzi* populations under study and three artificial clusters (all stocks from Sapini, all stocks from Quillacollo, and all TcI Bolivian stocks) and their 95% confidence intervals.

*F*_is_ among populations: *F*_is_ values per population and their 95% confidence intervals are shown in Figure 2. The *F*_is_ values were also examined by grouping the most adjacent populations, Sap-Sap with Sap-Cosi (1.9 km apart), Qui-Urk with Qui-Bsia (3.3 km apart), and all the populations (overall). *F*_is_ varied from −0.08 (Qui-Urk population) to 0.29 (overall). Considering the significance using the Bonferroni correction (BC), none of the *F*_is_ were significant (H_0_ not rejected, see Table 3) except for the overall sample. As we know that BC may falsely accept H_0_, we also considered the *p*-values without BC: here H_0_ is rejected with α = 1% within the “overall” sample and for only one sample grouping two local populations “overall Sapini” and was not rejected in all the local populations. Consequently, the decisions about panmixia were rejection for the “overall” sample, ambiguous for “overall Sapini,” and no rejection for all local populations (Table 3).

**Table 3:** Analysis of *F*_is_, disequilibrium linkage (LD) and repeated multilocus genotypes (MLGs) of the 79 *Trypanosoma cruzi* strains isolated from six potentially panmictic populations.

Populations	Overall	Luribay	Mecapaca	Overall Sapini	Sap-Sap	Sap-Cosi	Overall Quillacollo	Qui-Urk	Qui-Bsia
Sample size	79	6	11	46	27	19	16	12	4
Statistical tests of HWE based on *Fis* statistics									
Real *p*-value without BC^(1)^	0.006**	0.0371*	0.0182*	0.0075**	0.2243^NS^	0.0148*	0.0178*	0.3954^NS^	0.1096^NS^
Signification with BC^(2)^	**	NS	NS	NS	NS	NS	NS	NS	NS
Decision about H_0_	reject H_0_	no reject H_0_	no reject H_0_	*ambiguous*	no reject H_0_	no reject H_0_	no reject H_0_	no reject H_0_	no reject H_0_
Statistical tests of LD									
Ratio signif. / total^(3)^	16/28**	0/21^NS^	2/21^NS^	11/28**	7/28**	1/28^NS^	1/10^NS^	0/6^NS^	0/3^NS^
I_A_^(4)^	0.25**	-0.05^NS^	0.39*	0.31**	0.56**	0.002^NS^	0.63**	-0.08^NS^	1.31^NS^
ȓ_d_^(5)^	0.04**	-0.009^NS^	0.07*	0.05**	0.08**	0.0003^NS^	0.20**	-0.03^NS^	0.68^NS^
Decision about H_0_	reject H_0_	no reject H_0_	*ambiguous*	reject H_0_	reject H_0_	no reject H_0_	*ambiguous*	no reject H_0_	no reject H_0_
Statistical tests of repeated MLG									
No. of different MLGs	68**	6^NS^	10^NS^	43*	24*	19^NS^	9**	6^NS^	3^NS^
Maximum frequency of MLG	5**	1^NS^	2^NS^	2^NS^	2^NS^	1^NS^	5^NS^	5^NS^	2^NS^
Decision about H_0_	reject H_0_	no reject H_0_	no reject H_0_	*ambiguous*	*ambiguous*	no reject H_0_	*ambiguous*	no reject H_0_	no reject H_0_

Results of statistical tests and decisions about H_0_ (reject or not reject panmixia). For all tests: NS or ^NS^ = not significant; * = significant at 5% risk; ** = significant at 1% risk ^(1)^ p-value for *F*_is_ within samples without Bonferroni correction (BC); ^(2)^ significance of the test with BC; ^(3)^ Ratio: significant loci pairwise comparisons / total comparisons, tested by the binomial test with R program; ^(4)^ Value of index of association; ^(5)^ Value of ȓd index.

Linkage disequilibrium (LD): three parameters were tested: (i) the proportion of significant LD tests over the total number of comparisons by pairs of loci, using the binomial test, (ii) the association index (I_A_), a direct measure of LD, and (iii) a special index (ȓ_d_) derived from I_A_. These indices and their associated significance are given in Table 3. Of the six local populations under study, H_0_ was not rejected in four of them (Luribay, Sap-Cosi, Qui-Urk and Qui-Bsia); two results were ambiguous (Mecapaca and overall Quillacollo) and three rejected H_0_ (Overall, Overall Sapini and Sap-Sap).

Repeated multilocus genotypes: We tested two parameters, the number of different MLGs and the maximum frequency of the most repeated MLG. The results showed (Table 3) that H_0_ is rejected in only one sample (Overall), not rejected in five populations (Luribay, Mecapaca, Sap-Cosi, Qui-Urk, and Qui-Bsia) and ambiguous in three populations (Overall Sapini, Sap-Sap, and Overall Quillacollo).

Considering only the six potentially panmictic populations under study, in four of them (Luribay, Sap-Cosi, Qui-Urk, and Qui-Bsia) the decisions for *F*_is_, LD, and MLG were “no rejecting H_0_” , while in the two others (Mecapaca and Sap-Sap) contradictory results were observed between the different tests of panmixia. Nevertheless, for the only *F*_is_ tests within the populations from Luribay and Sap-Cosi, there is a potential risk of type II error

## Discussion

### Likely panmixia in several *T. cruzi* populations isolated from wild *T. infestans*

As previously recommended [25], we used three classes of classical population genetics parameters to study the mode of reproduction (i.e., Hardy-Weinberg equilibrium, linkage equilibrium, and presence of repeated MLG) and we showed that in four out of six potentially panmictic *T. cruzi* populations (Luribay, Sap-Cosi, Qui-Urk, and Qui-Bsia) sampled in restricted areas, true panmixia cannot be excluded. In the case of Luribay and Sap-Cosi, the *F*_IS_ tests required the Bonferroni correction to lead to the decision “no rejecting Ho”, carrying a high risk of Type II error. However, as Qui-Bsia has a small size (N = 4) and that we cannot rule out a statistical type II error in this case, we must consider only three panmictic populations Luribay, Sap-Cosi, and Qui-Urk. For the two other populations, the tests gave contradictory results in Sap-Sap and ambiguous results for LD tests in Mecapaca, which appears “more panmictic” than Sap-Sap. In this case we could infer a lack of power of the tests to explain these results; nevertheless, and except for Qui-Bsia where the sample size is very small, a high β error value (type II error) is unlikely because for comparable population sizes the tests can reject or not reject H_0_. Moreover, multiplying the tests decreases the probability of type II error and increases the power of the test. Hence we can consider that no rejection of H_0_ is equivalent to accepting panmixia, possibly except for Qui-Bsia. Moreover, it is interesting to note that the tests were very sensitive to the Wahlund effect (sampling from heterogeneous populations): when we grouped all six populations (overall), all the tests became highly significant, proving the heterogeneity between populations at the regional level. This was true to a lesser extent for overall Sapini and overall Quillacollo, showing a genetic structure at a very low geographic scale (a few kilometers).

### Role of sympatry and sampling design

To test panmixia, the first condition is natural sympatry; indeed, a nonsympatric sample may lead to genetic structuring and generate a Wahlund effect and consequently a false rejection of H_0_. As nobody knows precisely what sympatry means for this parasite, we picked up the populations within a very small area, not more than 1 ha, in which the triatomes and mammal hosts are assumed to move enough to allow parasite transmission from one host to another and hence generate opportunities for genetic exchanges; we named these populations “potentially panmictic” and tested them. Consequently, in such populations, when H_0_ is not rejected, and excluding a type II error discussed above, we can consider, a posteriori, that these populations were truly sympatric. Inversely, when H_0_ is rejected by some tests, as is the case for the Sap-Sap population and to a lesser extent for Mecapaca, a Wahlund effect due to a hidden genetic structure (itself possibly due to a lack of sympatry) could be inferred. Interestingly, when we analyzed the microsatellite data by the software Structure [40], we showed the presence of two distinct genomes in only Sap-Sap and Mecapaca, hence a hidden genetic structure, which can explain the rejection of H_0_ for some tests within these two populations (data not shown). Meanwhile for these two populations, choosing between the two alternative hypotheses (i.e., lack of sympatry or presence of some extent of clonality) is almost impossible. Sampling in areas that are not actually sympatric may therefore result in falsely rejecting H_0_. Inversely, as previously stated by others [41], selecting only one individual per subpopulation and pooling each of them into an artificial population generates misleading patterns and false conclusions regarding the mode of reproduction, in particular a significant reduction of LD and modified HW equilibrium, sometimes giving an erroneous picture of the recombining organism despite a high level of clonality. Obviously, our sampling method did not fit this pattern and consequently absence of H_0_ rejection cannot be attributed to this sampling bias. All these remarks emphasize the importance of sampling design to test the hypothesis under study, for example here, to test panmixia, we need potential sympatric areas, not allopatric areas.

### Clonality versus recombination in *T. cruzi* *species*

Since the pioneering studies using isoenzymes [6], *T. cruzi* has been considered by most authors to have a basically clonal population structure, with occasional bouts of genetic exchange or hybridization. These facts were confirmed on many occasions with other genetic markers and a clonal theory of parasitic protozoa was proposed [2,42] with the notable exception of *Plasmodium falciparum* in which sex occurs [43]; the theory was reaffirmed with both *Trypanosoma* and *Leishmania* genera [44] and extended to fungi bacteria and viruses in a recent review [45]. The question of determining whether sex occurs or not in *T. cruzi* is not trivial, nor needless. Because of a reduced or absent gene flow, clonality must have a major impact on the biological and medical properties of the parasites, which has been explored [46,47]. On the other hand, genetic exchanges can take different forms, the best known being hybridization that has been provoked *in vitro* [17] and has naturally occurred, playing a crucial role in *T. cruzi* evolution (generating new DTUs). It is generally admitted that two hybridization events have defined the population structure of *T. cruzi* [7], the first one very ancient, between TcI and TcII, leading to TcIII and TcIV, and the second one, recent, between TcII and TcIII, leading to TcV and TcVI. The *in vitro* hybrids showed a fusion of parental genotypes, loss of alleles, homologous recombination, and uniparental inheritance of kinetoplast maxicircle DNA [17], and it is accepted that natural hybridization might occur in a similar but contrasted way [48]. In addition to hybridization, many authors have reported incongruence between phylogenetic trees, which is generally a sign of recombination: for example 13,49, mitochondrial introgression [10,11] and even mitochondrial heteroplasmy (heterogeneous mitochondrial genomes in an individual cell) was demonstrated recently [16] using the promising mtMLST method (mitochondrial multilocus sequence typing), itself derived from the MLST method using nuclear genes [50]. The last way of genetic exchanges might be conventional recombination mechanisms, as in sexual diploids, which can be detected by the usual tools of population genetics (*F*_IS_, LD, etc., like here). Because we do not know the cytological mechanisms involved, we named these events “recombination-like” in order to differentiate them from the known genetic exchanges involving meiosis in sexual diploids. One of the first studies regarding this event [51], reported at one isoenzyme locus (phosphoglucomutase), observed homozygous and heterozygous frequencies almost identical to those predicted by the theoretical Hardy-Weinberg distribution in sylvatic TcI. Later, using microsatellites, some recombinations were suggested in a general clonal framework in sylvatic TcI over the endemic area [52], TcI in Ecuador [53], and TcIII [54]: in the latter, the authors could not effectively discriminate a recombination from a high genome-wide frequency of gene conversion. Finally, three recent studies emphasize the role of genetic exchanges and the extraordinary genome plasticity of *T. cruzi*, (i) using genomic CNV (copy number variation) [15]; (ii) another team [14] reported gross incongruence in Colombian TcI between nuclear and mitochondrial markers, mosaic maxicircle sequences, and the genetic resorting mechanism; (iii) other authors [55] showed that hybrid stocks contain haplotypes that are mosaics probably originating from intragenic recombination. In all these examples, it is worth noting that hybridization or introgression may occur between distant DTUs, whereas “recombination-like” events generally are intra-DTU, as shown in the present study. The “clonality or genetic exchanges” duality for *T. cruzi* has definitively became obsolete; this species obviously has used both mechanisms to evolve and probably to adapt to its multiple hosts, associated with an extraordinarily plastic genome shaped by clonal evolution and several kinds of genetic exchanges. The mode of reproduction of *T. cruzi* could oscillate between clonality and sexuality and the true questions are why, when, how, and to what extent *T. cruzi* recombines? Nevertheless, we agree with Tibayrenc and Ayala’s [45] definition of clonality as “restrained recombination on an evolutionary scale,” which has already been observed in *T. cruzi* since the same MLGs can be sampled at different times and in distant regions. The same authors stated that “recombination seems easier between closely related genotypes pertaining to the same near-clade in both fungi and parasitic protozoa”; this probably constitutes the most parsimonious explanation for the co-occurrence of recombination at restricted space / time levels and of clonality at larger space / time scales. Interestingly, in bacteria “the probability of acceptance of a recombination event decreases exponentially with genetic distance between the donor and recipient DNA” [56], which is an effect of sexual isolation in bacteria [57]; this could be true for *T. cruzi* and should be further investigated.

### Conclusion, limitations and warning

For the first time we report panmixia, notably through linkage disequilibrium statistics, in *T. cruzi* TcI populations isolated from wild *T. infestans* in Bolivia. In absence of additional studies involving other sylvatic vectors, it is not possible to associate panmixia with the sylvatic biotopes; further studies of panmixia should be conducted in other biotopes where parasites should be sympatric. As previously mentioned, “mixed clonal / sexual reproduction is nearly indistinguishable from strict sexual reproduction as long as the proportion of clonal reproduction is not strongly predominant” [30], so, although unlikely, we cannot exclude a certain level of clonality in these populations, even when all tests did not reject the panmixia hypothesis. Moreover, it is worth noting that the parasite strains used here were not cloned and some artifacts due to multiple infections could be a possible explanation for some contradictory results between the different tests. The *Leishmania* genome is aneuploid [58], every chromosome in every cell may be present in different ploidy states (monosomic, disomic, or trisomic). If this is the case for *T. cruzi*, as suspected [15], there could be a serious bias with all the codominant nuclear markers, particularly in the studies involving microsatellites: artificially decreasing *F*_is_ in the trisomic state (excess heterozygosity) and artificially increasing *F*_is_ in the monosomic state (excess homozygosity). Hence, all the *F*_is_ results should be interpreted with caution, especially when there is a substantial variance of *F*_is_ between loci. Moreover, *F*_is_ is not linearly related to the rate of clonal reproduction [59]. As stated above, the sampling strategy is crucial to confirm or reject these results in other natural contexts, avoiding sampling stocks that have a foreign origin because of passive transport by humans. For this purpose (to specify the mating system at the local scale), we recommend starting with a reduced time and space scale in order to avoid the Wahlund bias as much as possible, which does not hamper the opposite strategy previously proposed [45], “taking a birds-eye view of genetic variability over years and continents, from different hosts and ecosystems” to look at the evolution of the species over space and time.
